# Assessment of the welfare of breeding and boarding dog farms in the greater Cairo region: application of the Farm Quality Protocol (FQP)

**DOI:** 10.1186/s12917-024-04425-w

**Published:** 2025-03-03

**Authors:** Azhar F. Niazy, Basma M. Bawish, Mohamed Y. Matoock

**Affiliations:** Veterinary Hygiene and Management Department, Faculty of Veterinary. Medicine, Cairo University, Giza, 12211 Egypt

**Keywords:** Assessment, Boarding, Breeding, Dog, Protocol, Welfare

## Abstract

Millions of dogs are housed in facilities for industrial breeding and kennels worldwide. The care given to dogs in these facilities differs. The dog farm’s cramped conditions can have a negative influence on welfare. Therefore, monitoring and evaluation are crucial parts of the welfare protocol. There are currently no studies examining the welfare of dog farms (enclosed farms). The objective of this research was to assess an analytical description of dog farms and identify any potential risks to the welfare of these facilities. This was accomplished by using the Farm Quality Protocol (FQP), which is based on the Shelter Quality Protocol (SQP), to connect the elements influencing profits and outcomes by including 2,667 dogs in 20 dog farms of 22 different breeds, management systems, and facilities located in Greater Cairo (three cities in Egypt). There were descriptive and logistic regression analyses done. Key findings indicated that the score for body condition (BCS) was substantially connected with the type of diet as well as exercise. It has been demonstrated that the type of floor and its suitability affect the likelihood of discovering skin lesions. Clean bedding materials were substantially associated with a lower likelihood of seeing filthy or wet dogs. The abnormal behaviours, such as anxiety, were shown to be much higher when there was no enrichment in the facilities. In addition, the feeding schedule was linked to the presence of diarrhea. The shelter protocol was successful in recognizing welfare risks related to farm management and the environment. When these dangers are identified, there is a chance for improvement to be made, reducing the risks and enhancing the welfare of dog farms.

**Supplementary Information:**

The online version contains supplementary material available at 10.1186/s12917-024-04425-w.

## Background

Depending on the situation, different people may hold varying views regarding the well-being of dogs kept in kennels [1]. The public is concerned about dog welfare [2].

According to [3, 4], industrial dog breeds have raised a variety of welfare and ethical issues in recent years. Concerns about raising dogs in subpar settings without considering their demands for behaviour and physical health are frequently voiced.

The Farm Quality Protocol (FQP), which was based on the Protocol of Shelter Quality (SQP), was designed to assess the general welfare level of dog farms while upholding the standards and applicability [5]. It is a method used to investigate animal welfare issues by pinpointing crucial elements of the farm setting and administration by closely observing how the animal reacts to its surroundings [6].

According to [7], the standard of welfare used a multi-functional strategy that was modeled after its protocols. This consortium focused on farm animals and implemented four welfare principles, namely “good food,” “good housing,” “good health, and “appropriate behaviour.“.

Every principle consists of several welfare standards [8].

Group housing has become increasingly common, and care is being given to exercise, play, and socializing [9]. Single housing, on the other hand, is still often used in the rescue context, mainly due to heightened concerns about aggression and disease transmission [10].

Several factors, such as an animal’s species, age, prior experiences, health, and physiological condition, might affect how it reacts to stress. When faced with a challenge or stressor, for example, one animal of the same species may see it as a slight to its welfare, but another animal in the same circumstance may not regard it as such [11]. So, rather than focusing on gatherings of dogs, it is crucial to monitor and assess each dog’s welfare individually. According [6, 12], the scientific community is becoming more and more eager to offer reliable and simple-to-use instruments for assessing the adaptability and well-being of dogs housed in tiny shelter settings.

The relationship between earnings and results also enables the study of a welfare hazard analysis [13]. Significant welfare issues with Commercial Breeding Establishments (CBEs) have been brought up, including the possibility that they won’t receive enough veterinary care, proper housing, enrichment, exercise, and socialization [14, 15].

The current study’s purpose was to focus on the significance and advancement of dog farm welfare.

## Materials and methods

### Subjects and facilities

This study was conducted on twenty dog farms (enclosure farms), representing 14 breeding farms and 6 boarding farms. The farm owners granted permission to visit the farms before the study began.

The farms are managed by private managers and located in the Greater Cairo region (Cairo, Giza, and Qalyubia cities) in Egypt (Table 1).

**Table 1:** Number of dog farms selected from the greater Cairo region in Egypt and regions’ geographical areas

Egyptian region	Geographical area	Selected farms (*n*)
Cairo	Northeastern Egypt	3
Giza	*West bank of the Nile opposite central Cairo*	12
Qalyubia	Lower Egypt	5
Total		20

This study was conducted on 2267 dogs, including 1081 male (48%), 1186 female (52%), representing twenty-two different breeds (Table 2), consisting of 1932 in breeding farms and 335 in boarding farms, housed in 1565 different kennels.

There was a huge difference in the number of dogs used in the study as breeding farms were 14 farms, and 6 boarding farms.

In this study, a total of 1250 kennels were evaluated, including 855 double-sided and 395 one-sided kennels.

**Table 2:** Farm dog breeds, number, sex, and percentage

Breeds	*N*.	%	Sex			
			Male	%	Female	%
Armant	4	0.0%	2	50%	2	50%
Beagle	51	2%	27	53%	24	47%
Boxer	70	3%	37	53%	33	47%
Cane Corso	165	7%	71	43%	94	57%
Caucasian	2	0.0%	1	50%	1	50%
Chow chow	40	2%	15	38%	25	63%
Cocker	137	6%	66	48%	71	52%
Dalmatian	10	0.0%	4	40%	6	60%
Dobermann	20	1%	7	35%	13	65%
Dogo Argentinos	74	3%	34	46%	40	54%
German shepherd	704	31%	349	50%	355	50%
Golden retriever	78	3%	36	46%	42	54%
Great dane	59	3%	28	47%	31	53%
Griffon	190	8%	95	50%	95	50%
Husky	19	1%	10	53%	9	47%
Labrador	146	6%	71	49%	75	51%
Malinois	146	6%	62	42%	84	58%
Pekingese	107	5%	51	48%	56	52%
Pit Bull	80	4%	43	54%	37	46%
Presa Canario	5	0.0%	1	20%	4	80%
Rottweiler	123	5%	54	44%	69	56%
St. Bernard	37	2%	17	46%	20	54%
Total	2267		1081	48%	1186	52%

### Farms and the design of a general study

The current study was carried out from December 2019 to March 2023. The study’s questionnaire was developed with the intention of conducting in-person interviews with each farm owner to gather data. Forty (40) farm visits were made in order to collect data (Supplementary Information). During the visits, the farm managers had a positive and helpful approach.

This study used the “Farm Quality Protocol (FQP), which is based on the Shelter Quality Protocol (SQP)” [6] to measure the welfare of dog farms that breed and board dogs. hospitalized dogs, and sample size estimates did not take them into consideration. Three levels of evaluation were carried out (farm, kennel, and individual), featuring a selection of dogs and kennels for every level.

Depending on how many dogs each farm had, different dog populations were included in the sample size for individual evaluations. Only dogs who were at least six months old were taken into consideration for the study. Each kennel had one dog chosen at random. The number of kennels in the sample (one-side and double-side kennels) was established by considering both the type of kennel and the total number of dogs in the farm.

Data collection at the farm level reveals what kind of resources were available to the dogs and how they operated [6].

The sample size of the farm ranged from 11 to 100 dogs, totaling 1145 dogs which were evaluated individually (Table 3).

**Table 3:** Sample size compared to the total number of dogs kept in dog farms

Total dogs in farm	Assessed dogs
11	11
20	20
24	24
60–200	60
300+	100

All logistical and dynamic data on the farm (meal schedule, facilities available, etc.) was provided by the farm manager.

Management-Based Measure (MBMs) (such as farm demographics, feeding, dog exercise routine, etc.) were initially recorded at the farm level. Second, if not directed otherwise by the protocol, the evaluator conducted FQP at the kennel level while maintaining a distance of two meters from the fence and refraining from any animal interaction. At this stage, they observed RBMs and ABMs as key metrics.

Resources-Based Measure (RBMs) include things like the number of dogs per kennel, cleanliness, and space-allowed bedding sufficiency, as well as things such as jagged corners or perilous ridges inside the kennel or alongside the fence.

Animal-Based Measures (ABMs) observed in the kennel include dogs with coughing and diarrhea. Diarrhea is identified by the presence of liquid or moderate feces, along with evidence of fecal matter on a dog’s fur or perineal area. Additionally, individual ABMs such as dog hygiene and body condition score were documented independently (Table 4).

**Table 4:** Farm quality protocol measures associated with welfare principles and criteria

Principle	Welfare criteria	Welfare measure (type)	Level of assessment
Good Feeding	Absence of prolonged hunger	Body condition score (ABM)	Individual
		Feeding (MBM)	Farm
	Absence of prolonged thirst	Water supply (RBM)	Kennel
Good housing	Comfort around resting	Bedding (RBM)	Kennel
		Sharp edges (RBM)	Kennel
		Cleanliness of animals (ABM)	Individual
	Ease of movement	Space allowance (RBM)	Kennel
Good Health	Absence of injuries	Skin condition (poor coat) (ABM)	Individual
		Lameness	Individual
		Tail lesion (ABM) Body lesion (ABM)	Individual
	Absence of disease	Signs of diarrhoea (ABM)	Individual
		Ocular discharge (ABM)	Individual
			Individua
Appropriate behaviour	Expression of other behaviours	Abnormal behaviour (ABM)	Individual
		Pica (ABM) Coprophagia (ABM)	Individual
		Exercise (MBM)	Farm
	Good human-animal relationship	Reaction to human (ABM)	Individual

Types of measures defined in brackets: management-based measures (MBM); resources-based measures (RBM); animal-based measures (ABM). Measures were assessed according to three differing levels of assessment: the farm (evaluating the farm as a unit and all the animals within); the Kennel (evaluating the Kennel as a unit, considering all the dogs housed in the Kennel); and the individual (evaluating each animal as a unit)

### Visual physical health assessment

The physical health outcomes of dogs were evaluated through the application of the Body Condition Score (BCS), a nine-point scale. This system, established by [16] categorized lean dogs as having BCS scores ranging from one to three, while ideal dogs had BSC rankings between four and five. Overweight canines fell into categories six or seven, whereas obese pups attained a score greater than eight on the rating scale. Additionally, measurements for body and kennel cleanliness were conducted using another ranking feature called the Body Cleanliness Score (BC).

To further evaluate the dog’s physical condition during data collection, various observations were made, such as nasal discharge, ocular discharge, present lesions, wounds both visible around their tail, body, head, and legs, and in addition, diarrhea. External parasites’ presence or absence were also documented [17].

### Behaviour assessment

The ABMs were put through a brief behavoural test to see how the dogs responded to strangers. We split the test into two sections to capture the dogs’ responses. Step one was for the assessor to approach the outside barrier, stand in front of it for thirty seconds, and ignore the dog. The assessor kneeled and chatted to the dog respectfully for thirty seconds in the second step. An approach test (AT) for strangers was conducted in three steps by using the Field Instantaneous Dog Observation Tool (FIDO) in the kennel facility’s indoor area [18]. According to [19], this methodology does not quantify behaviour toward other dogs.

The dog’s responses at every stage were recorded using the Red-Yellow-Green (RYG) scoring system. Therefore, farm workers who are acquainted with their dogs have typically analyzed dogs’ behaviour against conspecifics.

Finally, after assessing the dogs’ behaviour towards strange people, the assessor used an emotional condition profile sheet to record the dogs’ emotional state, kennel by kennel. When the last kennel had been evaluated, the assessment was completed.

### Statistical analysis

For the purposes of the present study, a total of 1,145 dogs were assessed individually (475 male and 670 female). Adults (from 1 to 3 years) were 53%, young dogs (from 3 to 6 years) were 37%, and geriatrics (greater than or equal to 7 years of age) were 10%.

A descriptive analysis was designated to explore the variation of measures across farms and was conducted using SPSS (SPSS 24.0 software; SPSS Inc., Armonk, NY, USA). The prevalence of ABMs and mean percentages of RBMs and MBMs were calculated. An exploratory univariate analysis was performed to evaluate the association between income and outcome variables (MBMs and RBMs) and ABM. Histograms were drawn by Graph Pad Prism Version 9.00 for Windows (Graph Pad Software, LLC, File Version 9.0.0.(121)). All results were expressed as means ± SD and the significance level was set at *P* ≤ 0.05.

### Statistical evaluation of welfare hazard identification

The association between several predictors (RBMs and MBMs set as independent variables) and different welfare outcomes (ABMs set as dependent variables) was highlighted using the logistic regression analysis.

## Results

**Fig. 1:**
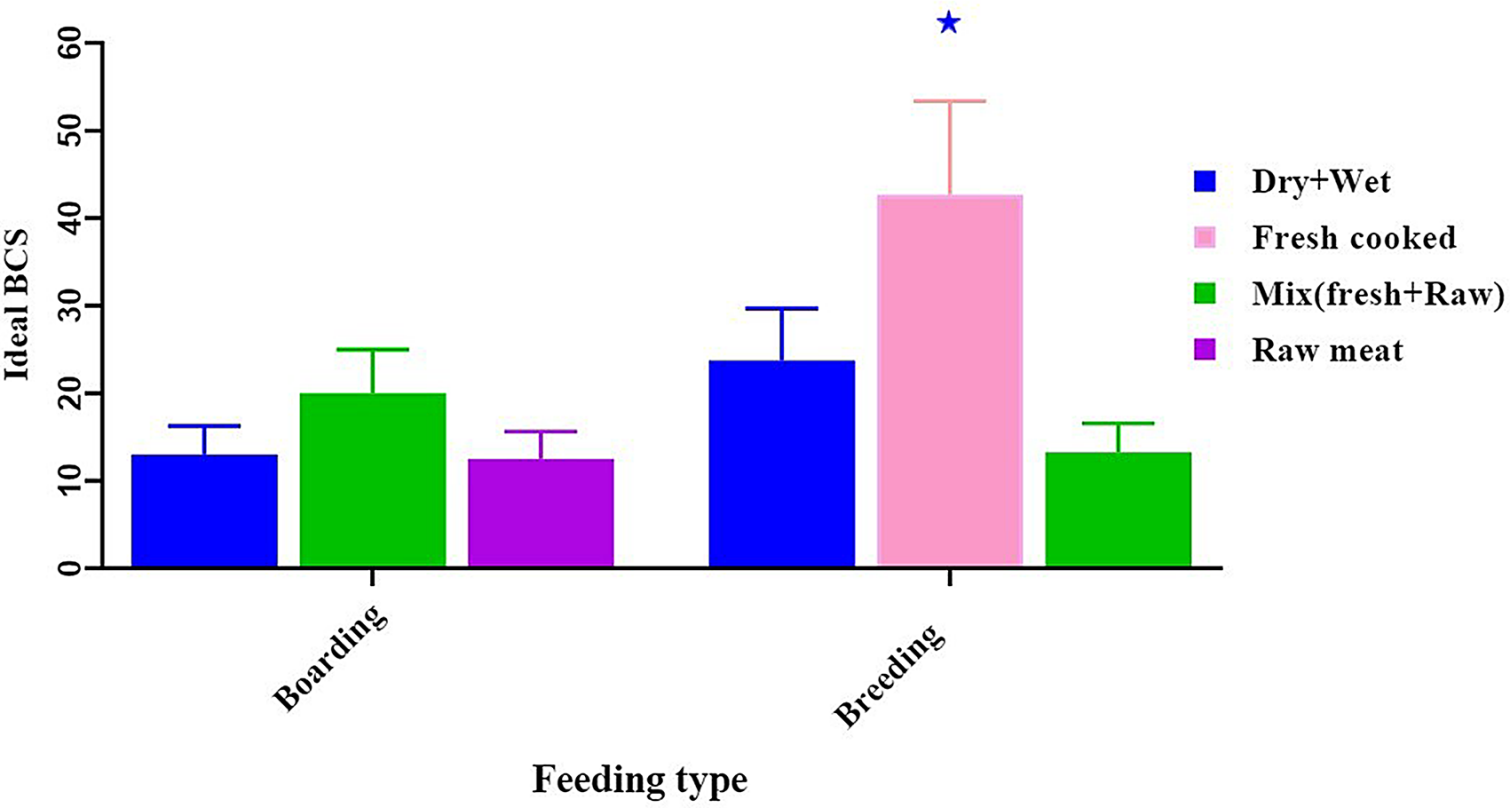
Effect of feeding type on a dog’s body condition score. *Mean significant difference at 0.05

**Fig. 2:**
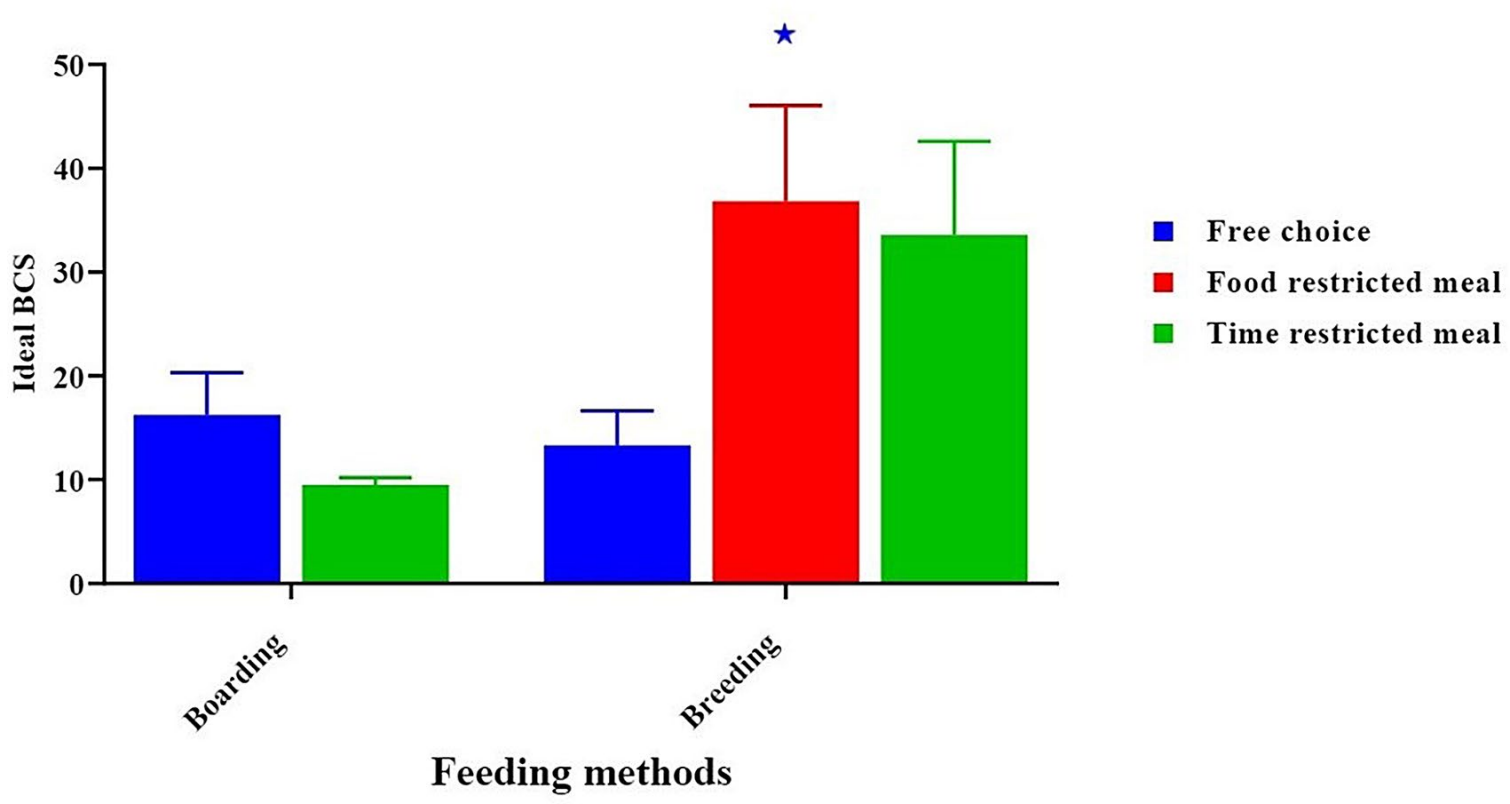
Effect of feeding methods on a dog’s body condition score. *Mean significant difference at 0.05

After doing an analysis of dog BCS, it was found that the probability of seeing a dog in an extremely thin body state was much higher, especially when dogs were given mixed food (fresh and raw) and canned or wet diet meals (30.00 ± 7.50, *p* < 0.05) in the boarding group. Compared to feeding fresh, eating cooked food increased the likelihood of seeing dogs with an optimal BCS. (42.67 ± 10.67, *p* < 0.05) in the breeding group. (Fig. 1), and free-choice feeding was positively associated with thin BCS (33.33 ± 5.77, *p* < 0.05) in the boarding group. whereas observing dogs with ideal BCS (36.83 ± 9.21, *p* < 0.05) in the breeding group, especially in the food-restricted meal method (Fig. 2).

**Fig. 3:**
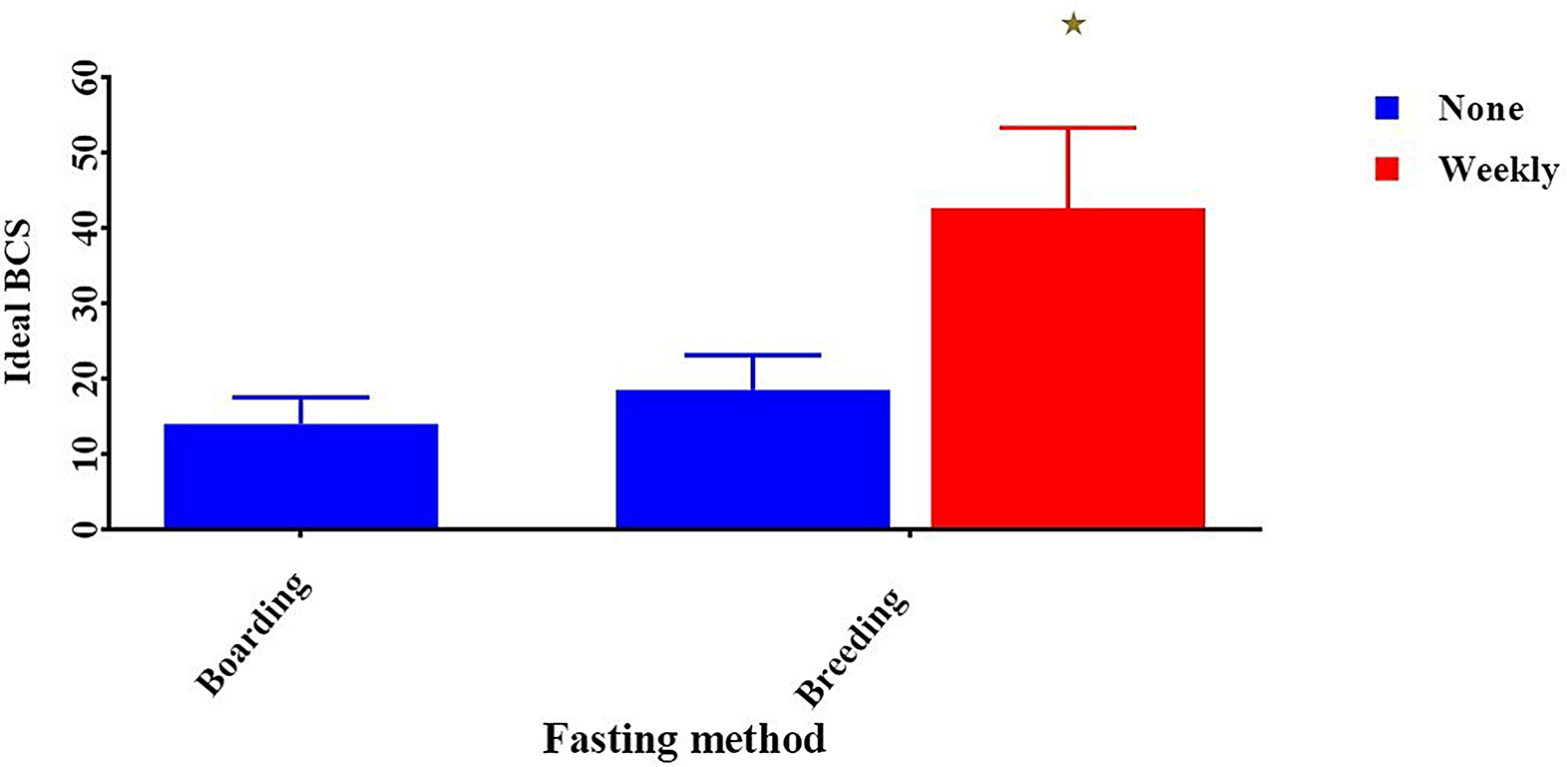
Effect of fasting method on a dog’s body condition score. *Means significant difference at 0.05

**Fig. 4:**
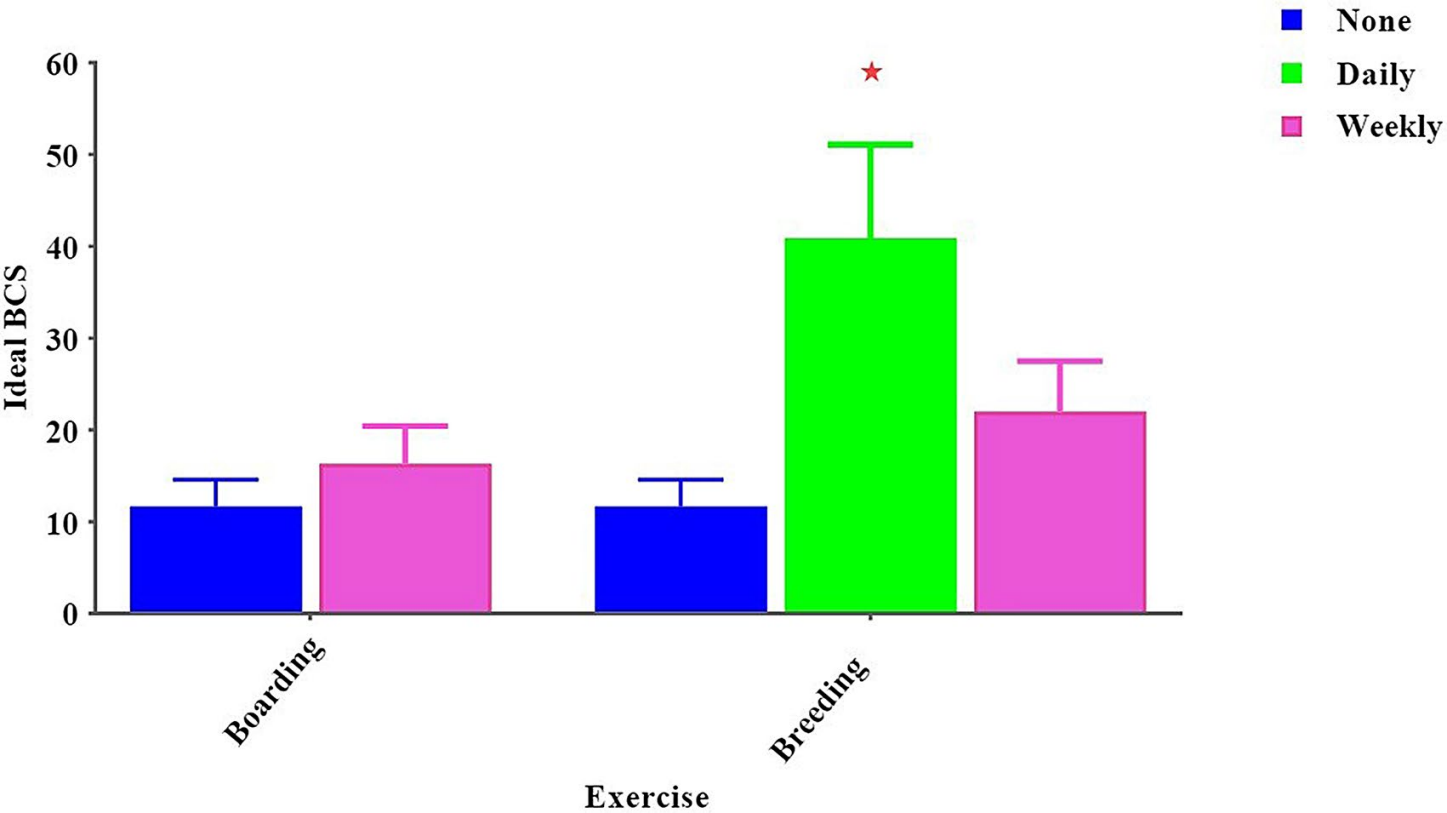
Effect of exercise method on a dog’s body condition score. *Means significant difference at 0.05

We also found fasting one day weekly has a very significant effect on ideal BCS dogs in the breeding group in comparison with boarding, which doesn’t make fasting day (42.67 ± 10.67 vs. 14.00 ± 3.5, *p* < 0.05). (Fig. 3**).** Additionally, daily exercise has a very significant effect on high ideal BCS “(40.86 ± 10.22, p < 0.05) in the breeding group. In contrast, boarding farms didn’t provide exercise or providing weekly exercise for their dogs. Observing dogs with thin BCS (11.67 ± 2.67, p < 0.05). (Fig. 4).

**Fig. 5:**
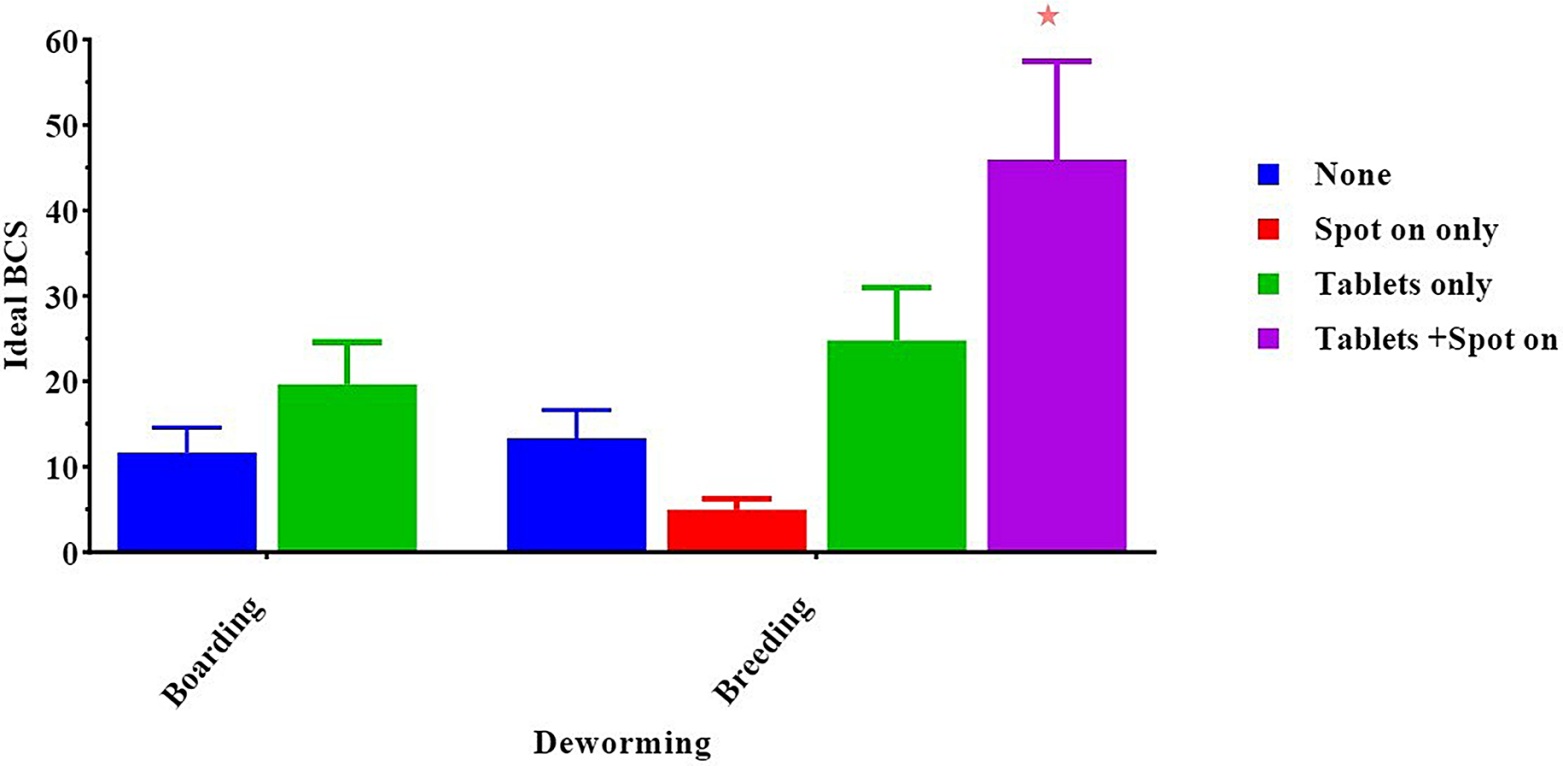
Effect of deworming on a dog’s body condition score. *Means significant difference at 0.05

Our result showed that deworming can also be considered a predictor of ideal BCS (46.00 ± 11.5, *p* < 0.05), especially when dogs are given both tablets and spot-on at breeding farms in contrast to boarding farms that use spot-on only (5.00 ± 1.25, *p* < 0.05) (Fig. 5).

**Fig. 6:**
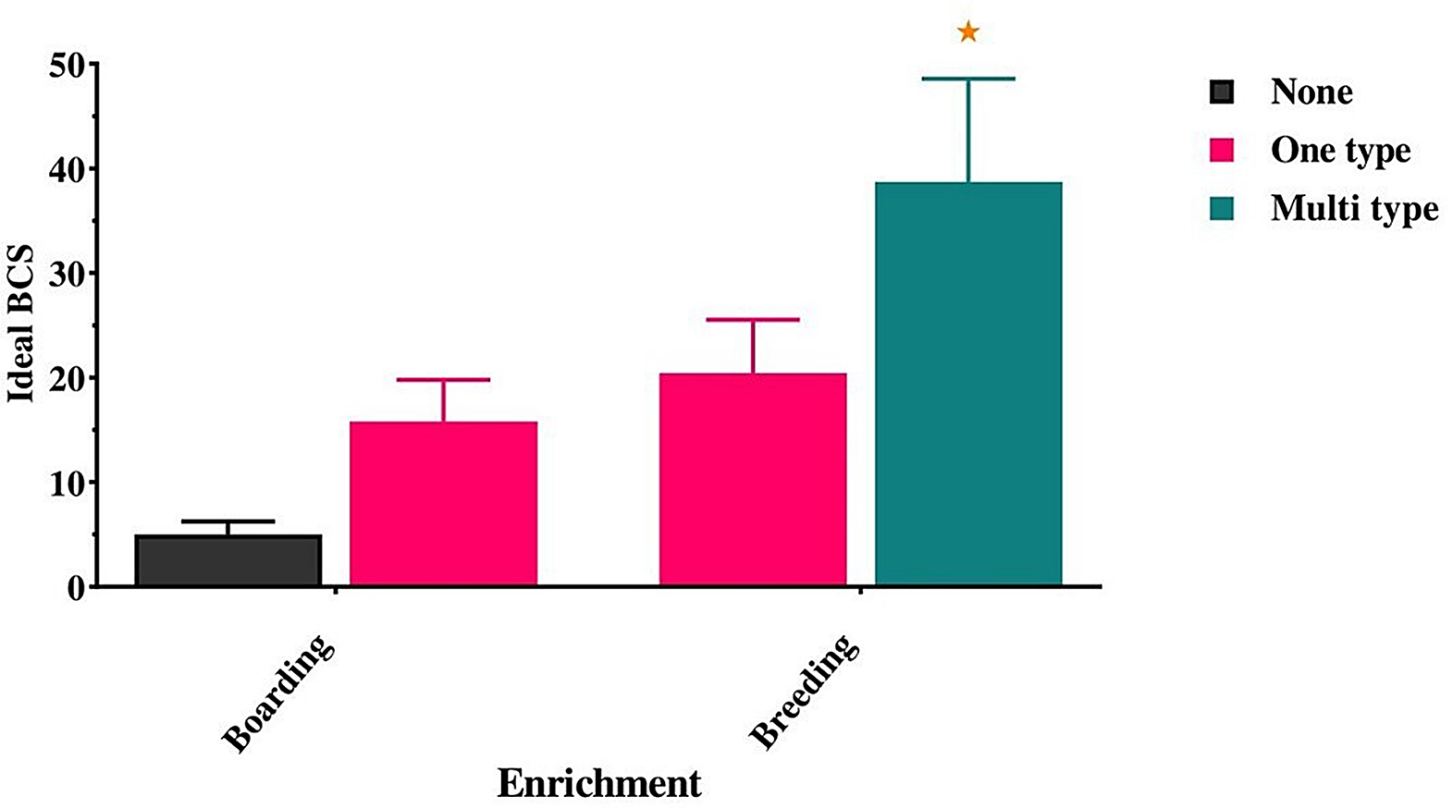
Effect of environmental enrichment on a dog’s body condition score. *Means significant difference at 0.05

Our result also found that environmental enrichment can also be considered a predictor of ideal BCS, especially when dogs are represented as multitype at breeding farms (38.71 ± 9.68) vs. boarding farms, which don’t offer any type of enrichment (5.00 ± 1.25, *p* < 0.05) (Fig. 6).

.

**Fig. 7:**
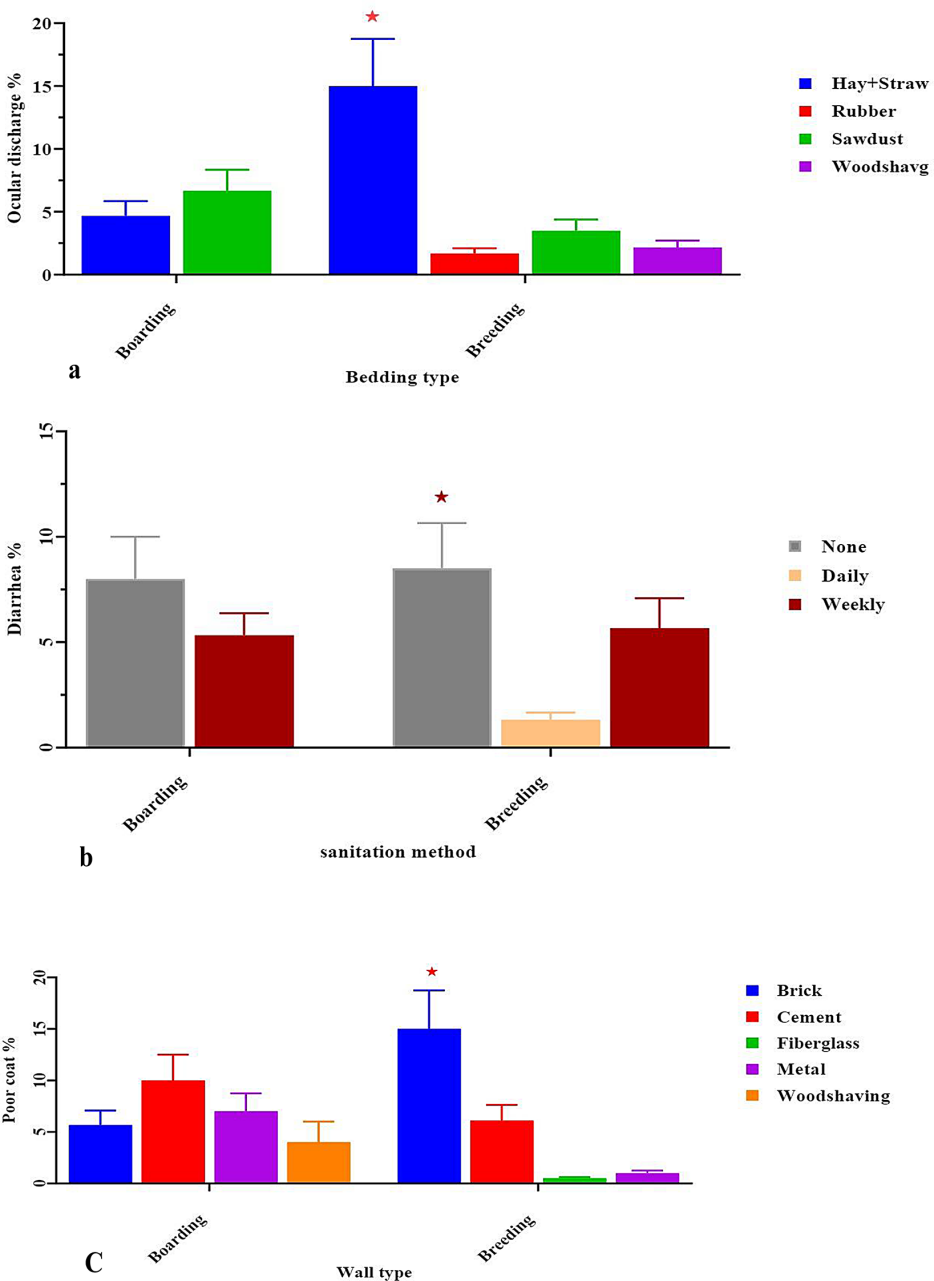
(**a**, **b**, **c**): Effect of some bedding, wall types and sanitation method on percentage of signs of diseases. *Means significant difference at 0.05

When analyzing body cleanliness, our results showed that signs of diarrhea and ocular discharge increased when Hay and Straw bedding types were presented per kennel (15.00 ± 3.75, *p* < 0.05). Also, no sanitation or deworming was a predictor of a high incidence of diarrhea (13.00 ± 3.25, *p* < 0.05). We also recorded that the brick and cement wall of the kennel was a predictor of a high incidence of poor coat, external parasites, foot injury, and lameness (15.00 ± 3.75, *p* < 0.05) (Fig. 7; a, b, c).

Our findings in the current study showed that rubber flooring has a significant impact on dog welfare by enhancing or detracting from dog comfort, safety, and cleanliness. In contrast to dogs kept on concrete floors, which are more prone to abrasions, alopecia, and lameness—especially in large breeds with smooth coats.

**Fig. 8:**
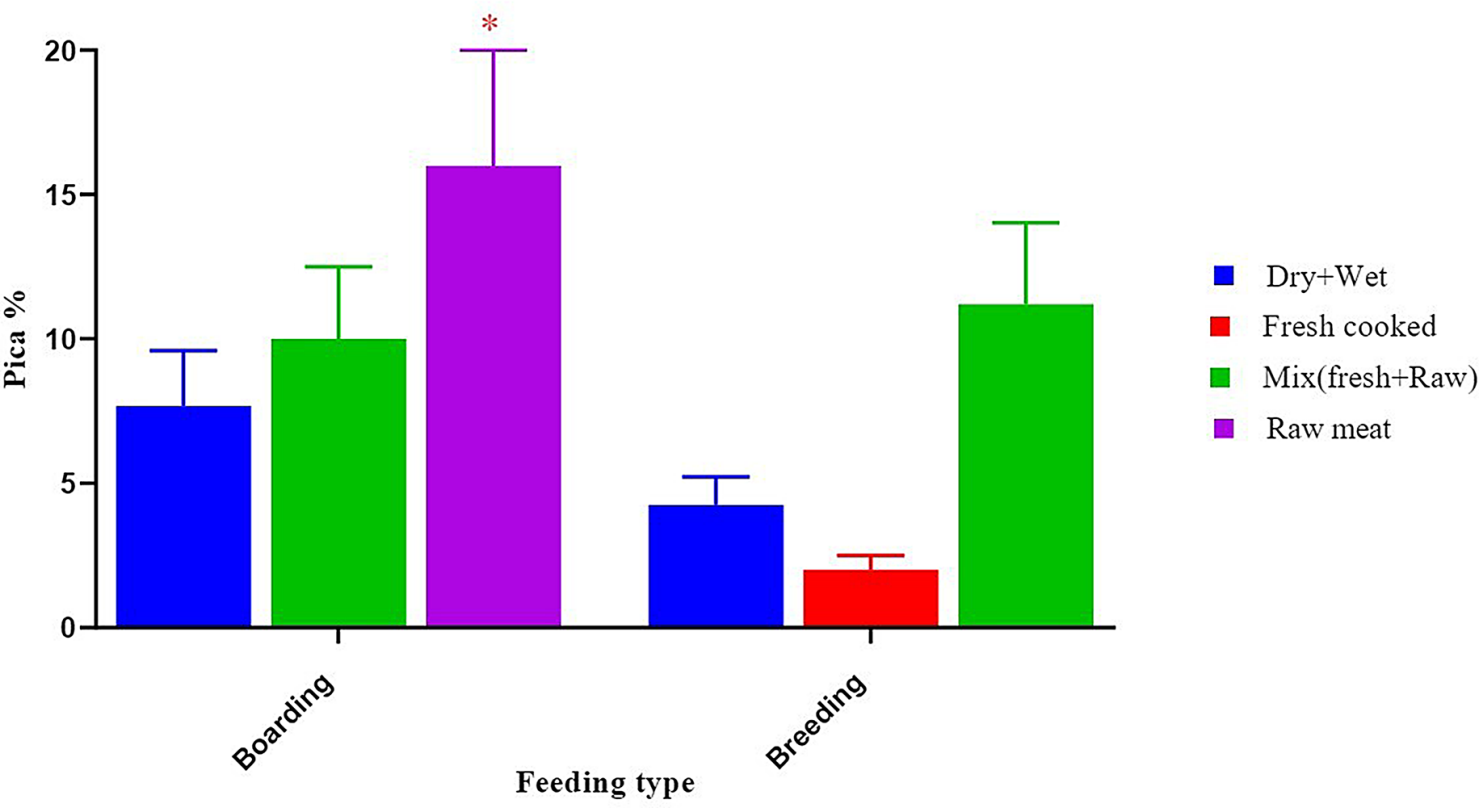
Effect of feeding type on percentage of pica. *Means significant difference at 0.05

**Fig. 9:**
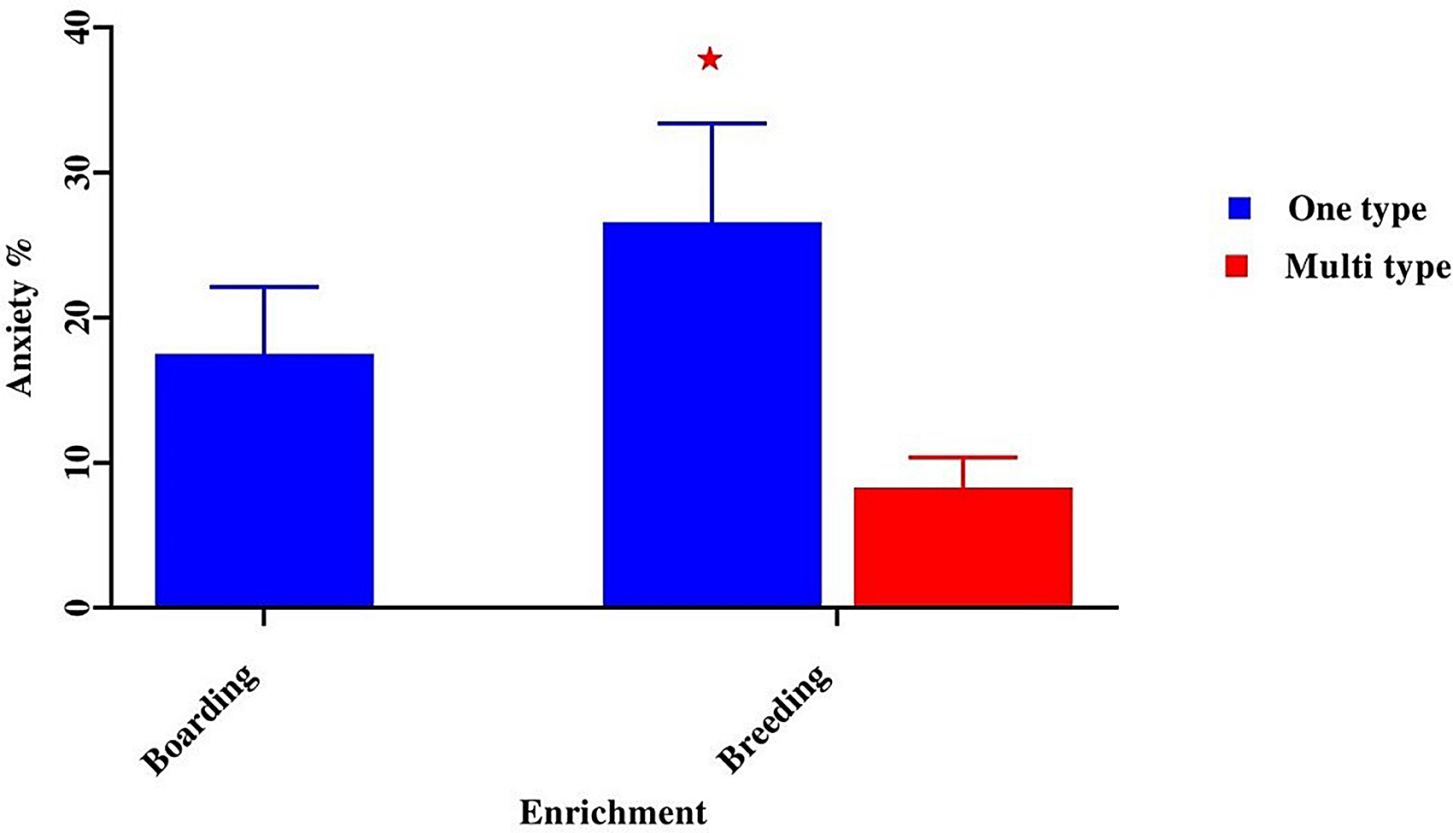
Effect of environmental enrichment on percentage of anxiety. *Means significant difference at 0.05

**Fig. 10:**
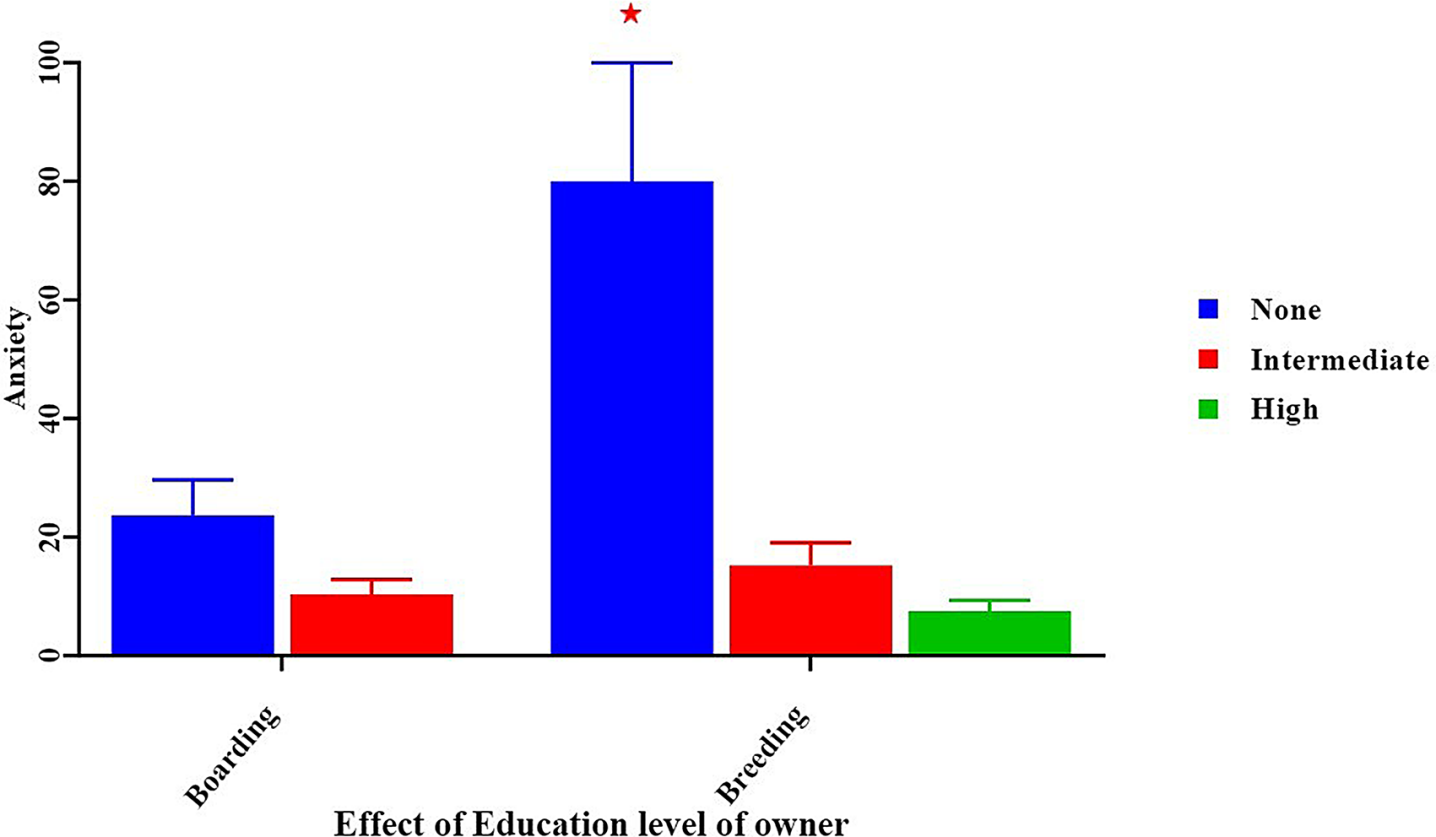
Effect of education level of owner on percentage of anxiety. *Means significant difference at 0.05

When analyzing some abnormal behaviour, our result recorded that diet type can be considered a predictor of pica and coprophagia, especially when dogs were fed a raw meat diet (16.00 ± 4.00, *p* < 0.05) (Fig. 8) and when dogs were not provided with any type or one type of enrichment (10.80 ± 2.70, *p* < 0.05) (Fig. 9). We recorded that the low or non-educational level of the owner and not providing any type or one type of enrichment were predictors of a high incidence of anxiety in dogs (80.00 ± 20.00 vs. 9.50 ± 2.38, *p* < 0.05) in contrast to a high education level (Fig. 10).

**Fig. 11:**
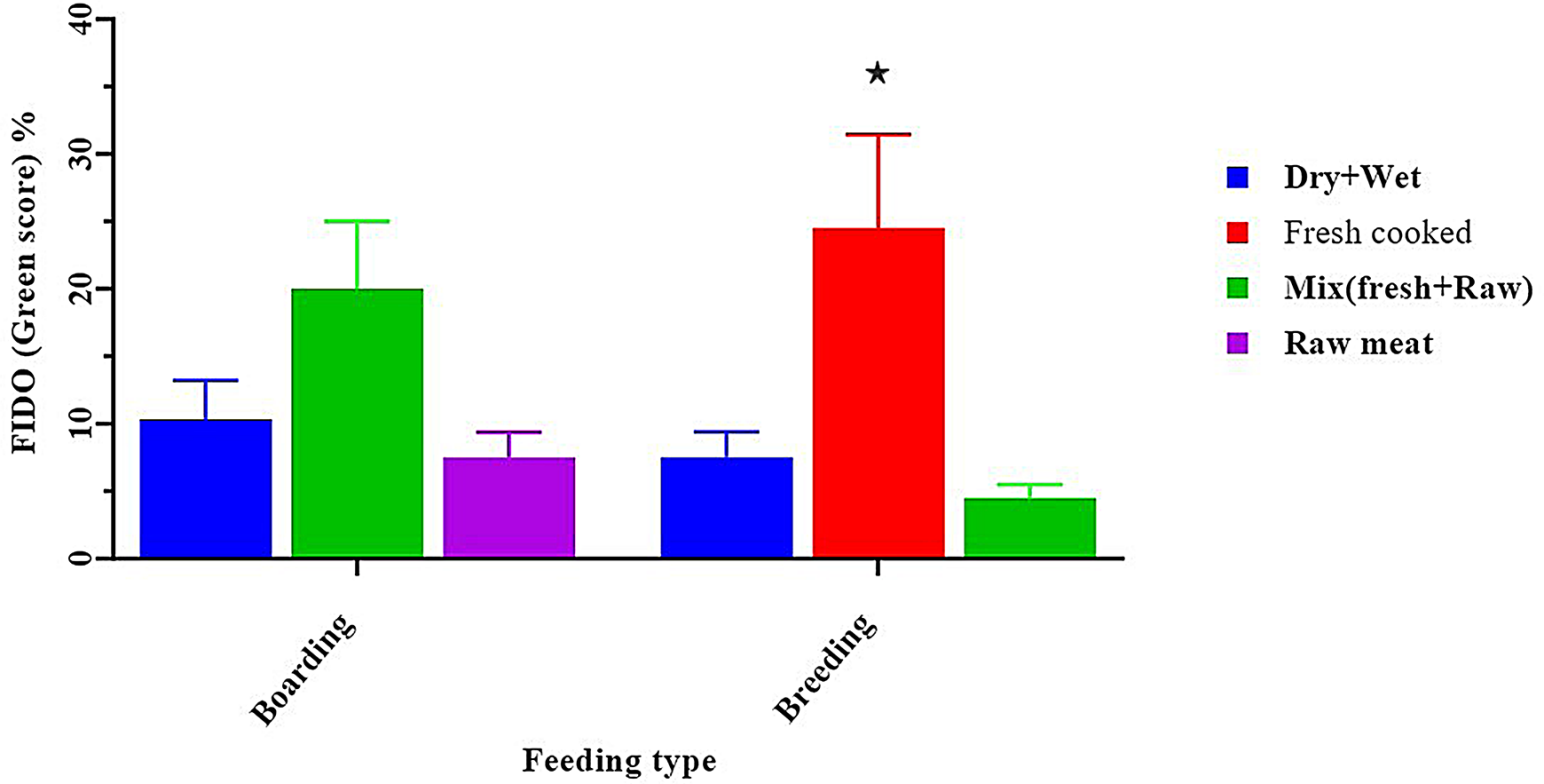
Effect of feeding type on percentage of green score of field instantaneous dog observation test. *Means significant difference at 0.05

By using the FIDO behavioural test to detect stranger-directed aggression, we observed a score. The probability of observing dogs with a rating in the red category was considerably higher (aggressive reflex) when fed on raw meat or dry and wet diet types (42.50 ± 11.75, *p* < 0.05), in spite of the fact that when fed on fresh cooked diet types, a dog recorded a green score (highly sociable reflex) (24.50 ± 6.95, *p* < 0.05) (Fig. 11). Our study found no association between the type of kennel and the observed aggressive behaviours in dogs.

Furthermore, our results showed some different factors had effects but were not significant: The Adequate space allowance effects on ideal BCS, for breeding farms (38.00 ± 9.5), for boarding farms (30.00 ± 7.50). The presence of sharp edge may occur some injury and lesion (tail, Body, lameness), for breeding farms (5.4 0 ± 1.35), for boarding farms (3.00 ± 0.75). The daily/weekly grooming effects on ideal BCS, for breeding farms (56.67 ± 14.17), for boarding farms 50.25 ± 10.56).

## Discussion

The findings of this study demonstrated that dogs fed raw meals (Raw Animal Product (RAP)) had thin body scores; this finding was consistent with a prior study [20. 21, 22, 23, 24, 25, 26], which demonstrated that dogs that are fed fresh food have better health than those who are fed commercial dry food [27], which corroborates these findings.

We found no correlation between a dog’s feeding frequency and health. We also acknowledge that free-fed dogs may ordinarily limit the amount of food they eat each day. This leads to compliance with [28]. According to the results of this study, fasting days or intermittent fasting lead to the highest dog body score. Several previous research have reported our findings by [29, 30].

It showed that dogs’ health and welfare are greatly enhanced in kennels by exercise, walks, and social environmental enrichment, with these findings supporting previous research [31, 32]. According to our research, giving dogs regular exercise helps to lessen dog prejudices. According to [33], this is verified.

Internal deworming, or endoparasiticides, have been shown to have positive benefits on dogs’ and their owners’ health in our research. This is in line with additional research that [34] completed, particularly with regard to the finding that dogs that have not had their worms removed had greater infection rates [35]. In line with past research, our findings also demonstrated that three or four annual deworming treatments do not offer total protection against endoparasites [35]. Our results align with previous research that has demonstrated the significance of deworming pregnant female dogs to prevent the spread of parasites from newborn puppies and the reactivation of larvae [36].

The results of external deworming (ectoparasiticides) have shown that controlling ectoparasites in dogs is crucial for the dogs’ particular health and welfare, as well as their general health and well-being. These findings are shown in a study by [37] and agree with previous studies showing that using ectoparasiticide boosted defenses against the spread of infections carried by arthropods [38–40].

Considering our outcomes, spot-on (ectoparasiticdes) was effective for the removal of ticks and fleas, as well as the prevention and treatment of demodex sarcoptic mange; this is in line with the results of [41, 42]. We also agreed with a new generation of Chewable Tablets for Dogs. The rate at which oral delivery occurs allows for the detection of flea activity two hours after oral administration [43, 44].

The results of the study’s investigation showed that incorporating different games into the surroundings of a dog farm, along with environmental enrichment approaches, may greatly enhance the dogs’ quality of life. The results of this investigation are consistent with those of the other prior studies [45, 46, 47].

The study’s findings demonstrated that inadequate bedding increases the danger of skin diseases in dogs, such as the presence of lesions, and cleanliness issues, such as a dirty or wet coat. Based on the findings of our investigation, the welfare of dogs should always come first when choosing the kind and caliber of bedding. This was found in a previous study [48]. Rubbers are the greatest kind of bedding, according to our research [49].

The results indicated that good hygiene and environmental disinfection can reduce the incidence of sickness. The results of previous studies conducted by [50–52] are entirely in line with our findings.

Our conclusion showed that a dog’s hair coat may be disheveled, matted, or tangled when there has been insufficient maintenance. Similar to the findings of [53], long-term matting of the hair can cause ischemic necrosis, underlying bone loss, and wrapping of the distal extremities by strangling the underlying tissue.

Research on the impacts of different factors is scarce in housing, like flooring [54], on dog welfare, despite studies having been carried out on topics such as how much and what kind of room is given to dogs and how environmental enrichment affects kennel surroundings [48, 55]. Furthermore, it doesn’t seem like any published research has examined these topics in relation to commercial breeding operations. The current study’s findings showed that rubber flooring significantly affects canine welfare by enhancing or impairing dog comfort, safety, and cleanliness. We also found that concrete, a more abrasive flooring surface than DCEM or POLY, is more frequently used to house larger-breed dogs. This might have led to an increase in alopecia and lameness cases [56].

We ensure in our result that there are no identical reasons for eating foreign body (FB), so we agree with previous studies by [57–59].

According to the results of our study, dogs who participated in an enrichment program significantly decreased abnormal behaviours such as anxiety. These results are in keeping with an earlier study carried out by [47] The current study’s findings are consistent with those of [10], who demonstrated that insufficient space in dog facilities leads to an increase in aberrant behaviors like anxiety. Our results imply that social interaction may be necessary for dogs kept in kennels to experience significant behavioral changes. This aligns with different research [60].

## Conclusions

Freshly cooked food, fasting one day a week, daily exercise, providing multiples of enrichment, deworming and a high educational level of owner result in good health, normal behaviour and so on the welfare of dogs.

The space allowances, daily grooming and presence of sharp edge had no significant effect on behaviour and welfare as well.

## Electronic supplementary material

Below is the link to the electronic supplementary material.

Supplementary Material 1

## Data Availability

No datasets were generated or analysed during the current study.

## Abbreviations

ABMs: Animal-Based Measures
BC: Body Cleanliness
BCS: Body Condition Score
FIDO: Field Instantaneous Dog Observation
FQP: Farm Quality Protocol
MBMs: Management-Based Measure
RBMs: Resources-Based Measure
RYG: Red-Yellow-Green
SQP: Shelter Quality Protocol
