# Supplementary material for: Assessment of the welfare of breeding and boarding dog farms in the greater Cairo region: application of the Farm Quality Protocol (FQP)
Source: BMC Vet Res. 2025 Mar 3;21:139. doi: 10.1186/s12917-024-04425-w (PMC11874446; doi:10.1186/s12917-024-04425-w)
Supplement: Supplementary file 1 — Supplementary Material 1 [file 12917_2024_4425_MOESM1_ESM.docx]

**Supplementary Information: Dog Farms Questionnaire**

| **Farm Information** | | | | | | | | | | | | | | | | | | | | | | | | |
| --- | --- | --- | --- | --- | --- | --- | --- | --- | --- | --- | --- | --- | --- | --- | --- | --- | --- | --- | --- | --- | --- | --- | --- | --- |
| **Name of farm** | | | | | | | | | | | | |  | | | | | | | | | | | |
| **Farm dimensions** | | | | | | | | | | | | | m^2^ | | | | | | | | | | | |
| **Name of owner** | | | | | | | | | | | | |  | | | | | | | | | | | |
| **Address** | | | | | | | | | | | | |  | | | | | | | | | | | |
| **City** | | | | | | | | | | | | |  | | | | | | | | | | | |
| **Phone/Mobile** | | | | | | | | | | | | |  | | | | | | | | | | | |
| **Number of employees/trainers/volunteers** | | | | | | | | | | | | | 00 Employees/ 00 Trainers | | | | | | | | | | | |
| **What the purpose of the farm?** | | | | | | | | | | | | | Boarding  Training  Breeding  Selling | | | | | | | | | | | |
| **Farm Management** | | | | | | | | | | | | | | | | | | | | | | | | |
| **Level of Education:** | | | | | | | | | | | |  | | | | | | | | | | | | |
| **Owner** | | | | | | | | | | | | Primary school  High school  Higher education | | | | | | | | | | | | |
| **Employees** | | | | | | | | | | | | Primary school  High school  Higher education | | | | | | | | | | | | |
| **Trainers** | | | | | | | | | | | | Primary school  High school  Higher education | | | | | | | | | | | | |
| **Knowledge of Farm Management** | | | | | | | | | | | | Poor  Good  Very good | | | | | | | | | | | | |
| **Facilities** | | | | | | | | | | | | | | | | | | | | | | | | |
| **General facilities:**  **General housing condition**  **Electric power**  **Water resource**  **Storage of food and/ or bedding**  **Facilities for caretaker’s cleanliness**  **Premises (building and grounds).** | | | | | | | | | | | | ☐ Poor  Fair ☐ Good  Very good  Yes  No  Surface water  Ground water  Yes  No  Yes  No  Yes  No | | | | | | | | | | | | |
| **Indoor Facilities:**  **Heating resource**  **Ventilation methods**  **Lighting resource**  **Interior surfaces**  **Drainage system** | | | | | | | | | | | | Electric space heaters  Fireplaces  Natural ventilation  Windows for ventilation  Spot ventilation  Natural Daylight  Fluorescent Lighting  Impervious  Not impervious  Surface Drainage  Subsurface drainage  Slope Drainage | | | | | | | | | | | | |
| **Outdoor facilities:**  **Protection from weather elements** | | | | | | | | | | | | **Cold weather**  Oil heater  Electric fireplace  **Hot weather**  Air-condition  Electric fan | | | | | | | | | | | | |
| **Enrichment** | | | | | | | | | | | | Provide a variety of toys  Play hide-and-seek  Go on outings  Set up group play sessions  Teach basic manners and life  skills  Keep a treat bucket handy  Help dogs with return-to-run  resistance  Offer a variety of smells and  sounds  Think outside the box:  Digging  Towel hide  Shaping games | | | | | | | | | | | | |
| **Housing** | | | | | | | | | | | | | | | | | | | | | | | | |
| **Type of housing** | | | | | | | | | | | Kennel  Cage  Pen | | | | | | | | | | | | | |
| **Total number of housed dogs** | | | | | | | | | | | Kennel Cages  Pens | | | | | | | | | | | | | |
| **Types of kennels** | | | | | | | | | | | Wire  Plastic  Wood  One-sided  Double-sided | | | | | | | | | | | | | |
| **Dimensions of kennel** | | | | | | | | | | | Length Width m | | | | | | | | | | | | | |
| **Dimensions of cages** | | | | | | | | | | | Length Width m | | | | | | | | | | | | | |
| **Dimensions of pen** | | | | | | | | | | | Length Width m | | | | | | | | | | | | | |
| **Dimensions of double-sided housing**  **Bedding** | | | | | | | | | | | Length Width m  Present  Absent  For puppies  Carton  Sawdust  wood shaving  Hay and strow for adult  Adequate  Inadequate | | | | | | | | | | | | | |
| **Floor**  **Roofs**  **Walls** | | | | | | | | | | | Concrete  Grass  gravel  Dirt  Plastic  Rubber  Sand  Flat  Gabled  Skillion  Wire  Wood  Metal  DIY chain link  Cement  Bricks  Fiberglass | | | | | | | | | | | | | |
| **Sharp Edges** | | | | | | | | | | | Yes  No | | | | | | | | | | | | | |
| **Are the kennels/cages/Pens Crowded?** | | | | | | | | | | | Yes  No | | | | | | | | | | | | | |
| **Is there a runway?**  **What is the type of runway?** | | | | | | | | | | | Yes  No  Natural grass  Wood chips  Gravel  Artificial grass  Paving stone | | | | | | | | | | | | | |
| **Cleanliness?**  **If Dirty?** | | | | | | | | | | | Clean  Dirty  Faeces  Urine  Others | | | | | | | | | | | | | |
| **Where are the dogs during cleaning?** | | | | | | | | | | |  | | | | | | | | | | | | | |
| **Total numbers of dogs** | | | | | | | | | | | 00 dogs | | | | | | | | | | | | | |
| **Registration** | | | | | | | | | | | Applied  Not Applicable | | | | | | | | | | | | | |
| **Dog Microchip ID** | | | | | | | | | | | Applied  Not Applicable | | | | | | | | | | | | | |
| **Total numbers kennel/cage/Pen** | | | | | | | | | | | Kennel  Cage  Pen | | | | | | | | | | | | | |
| **Numbers of dogs Per Kennel/cage/pen** | | | | | | | | | | | 00 dog per kennel/Cag  00 puppies per Pen | | | | | | | | | | | | | |
| **Total number of breeds** | | | | | | | | | | | | | | | | | | 00 Breeds | | | | | | |
| **Number of each breed** | **Adult** | | | | | | | | | | | | | | | | | | | | **Puppies** | | | |
|  | **Male** | | **Female** | | | | | | | | | | | | | | | | | | **Male** | | **Female** | |
|  |  | | **Pregnant** | | | | | | | | | **Non-Pregnant** | | | | | | | **Nursing** | |  | |  | |
| **Armant** |  | |  | | | | | | | | |  | | | | | | |  | |  | |  | |
| **Beagle** |  | |  | | | | | | | | |  | | | | | | |  | |  | |  | |
| **Boxer** |  | |  | | | | | | | | |  | | | | | | |  | |  | |  | |
| **Cane Corso** |  | |  | | | | | | | | |  | | | | | | |  | |  | |  | |
| **Caucasian** |  | |  | | | | | | | | |  | | | | | | |  | |  | |  | |
| **Chow Chow** |  | |  | | | | | | | | |  | | | | | | |  | |  | |  | |
| **Cocker** |  | |  | | | | | | | | |  | | | | | | |  | |  | |  | |
| **Dalmatian** |  | |  | | | | | | | | |  | | | | | | |  | |  | |  | |
| **Dobermann** |  | |  | | | | | | | | |  | | | | | | |  | |  | |  | |
| **Dogo Argentinos** |  | |  | | | | | | | | |  | | | | | | |  | |  | |  | |
| **German Shepherd** |  | |  | | | | | | | | |  | | | | | | |  | |  | |  | |
| **Golden Retriever** |  | |  | | | | | | | | |  | | | | | | |  | |  | |  | |
| **Great Dane** |  | |  | | | | | | | | |  | | | | | | |  | |  | |  | |
| **Griffon** |  | |  | | | | | | | | |  | | | | | | |  | |  | |  | |
| **Husky** |  | |  | | | | | | | | |  | | | | | | |  | |  | |  | |
| **Labrador** |  | |  | | | | | | | | |  | | | | | | |  | |  | |  | |
| **Malinois** |  | |  | | | | | | | | |  | | | | | | |  | |  | |  | |
| **Pekingese** |  | |  | | | | | | | | |  | | | | | | |  | |  | |  | |
| **Pit Bull** |  | |  | | | | | | | | |  | | | | | | |  | |  | |  | |
| **Presa Canario** |  | |  | | | | | | | | |  | | | | | | |  | |  | |  | |
| **Rottweiler** |  | |  | | | | | | | | |  | | | | | | |  | |  | |  | |
| **St. Bernard** |  | |  | | | | | | | | |  | | | | | | |  | |  | |  | |
| **Age of each breed** | | | | **0-2****MTH** | | | | | | | | **3-6** **MTH** | | **1-2****YRS** | | | | | | **3-6****YRS** | | **6-10** **YRS** | | **+ 11****YRS** |
| **Armant** | | | |  | | | | | | | |  | |  | | | | | |  | |  | |  |
| **Beagle** | | | |  | | | | | | | |  | |  | | | | | |  | |  | |  |
| **Boxer** | | | |  | | | | | | | |  | |  | | | | | |  | |  | |  |
| **Cane Corso** | | | |  | | | | | | | |  | |  | | | | | |  | |  | |  |
| **Caucasian** | | | |  | | | | | | | |  | |  | | | | | |  | |  | |  |
| **Chow Chow** | | | |  | | | | | | | |  | |  | | | | | |  | |  | |  |
| **Cocker** | | | |  | | | | | | | |  | |  | | | | | |  | |  | |  |
| **Dalmatian** | | | |  | | | | | | | |  | |  | | | | | |  | |  | |  |
| **Dobermann** | | | |  | | | | | | | |  | |  | | | | | |  | |  | |  |
| **Dogo Argentinos** | | | |  | | | | | | | |  | |  | | | | | |  | |  | |  |
| **German Shepherd** | | | |  | | | | | | | |  | |  | | | | | |  | |  | |  |
| **Golden Retriever** | | | |  | | | | | | | |  | |  | | | | | |  | |  | |  |
| **Great Dane** | | | |  | | | | | | | |  | |  | | | | | |  | |  | |  |
| **Griffon** | | | |  | | | | | | | |  | |  | | | | | |  | |  | |  |
| **Husky** | | | |  | | | | | | | |  | |  | | | | | |  | |  | |  |
| **Labrador** | | | |  | | | | | | | |  | |  | | | | | |  | |  | |  |
| **Malinois** | | | |  | | | | | | | |  | |  | | | | | |  | |  | |  |
| **Pekingese** | | | |  | | | | | | | |  | |  | | | | | |  | |  | |  |
| **Pit Bull** | | | |  | | | | | | | |  | |  | | | | | |  | |  | |  |
| **Presa Canario** | | | |  | | | | | | | |  | |  | | | | | |  | |  | |  |
| **Rottweiler** | | | |  | | | | | | | |  | |  | | | | | |  | |  | |  |
| **St. Bernard** | | | |  | | | | | | | |  | |  | | | | | |  | |  | |  |
| **Dogs Colour** | | | | | | | | | | | | | | | | | | | | | | | | |
| White Grey Tan Black      Red Brindle Merle Brown      Gold Blue Cream Yellow | | | | | | | | | | | | | | | | | Red and White    Black and Brown    Black and Tan    Tri-Colour | | | | | | | |
| **Medical Health and Physical Well Being** | | | | | | | | | | | | | | | | | | | | | | | | |
| **What is the Dogs Body Conditions in General?** | | | | | | | | | | | | | | | | Poor  Good  Very good | | | | | | | | |
| **Body Condition Score (BCS)** | | | | | | | | | | | | | | | | **Total Dogs:**  Too Thin (from 1 to 3)  Ideal (5)  Too Heavy (from 7 to 9) | | | | | | | | |
| **Medical and Behavioural Observations** | | | | | | | | | | | | | | | | | | | | | | | | |
| **Do you notice any obviously sick dogs?** | | | | | Yes  No | | | | | | | | | | | | | | | | | | | |
| **Body Cleanliness (Percent of the body covered in debris)** | | | | | 0=0  1=1-25%  2=26-50%  3=51-75%  4= >76% | | | | | | | | | | | | | | | | | | | |
| **Nasal Discharge**  **Ocular discharge**  **Sneezing**  **Coughing**  **Diarrhea**  **Vomiting**  **Missing fur or poor coat**  **Wounds, sores or lesions**  **Lameness**  **Extremal Parasite**  **Dystocia**  **Toxicity** | | | | | Present  Absent  Present  Absent  Present  Absent  Present  Absent  Present  Absent  Present  Absent  Present  Absent  Present  Absent  Present  Absent  Present  Absent  Present  Absent  Present  Absent | | | | | | | | | | | | | | | | | | | |
| **Do you notice any abnormal behaviours in dogs?** | | | | | Yes  No | | | | | | | | | | | | | | | | | | | |
| **Aggression:**  **a) Stranger-directed aggression**  **b) Owner-directed aggression**  **c) Dog-directed aggression**  **d) Maternal aggression** | | | | | Present  Absent  Present  Absent  Present  Absent  Present  Absent | | | | | | | | | | | | | | | | | | | |
| **Dog Fears and Phobias:**  **Fear of Thunder**  **Fear of Fire work**  **Fear of Being left Alone (Sep. Anxi.)**  **Fear of the Veterinarian.**  **Fear of Riding in the Car**  **Fear of People**  **Fear of Strangers**  **Fear of Children**  **Fear of Specific Objects** | | | | | Present  Absent  Present  Absent  Present  Absent  Present  Absent  Present  Absent  Present  Absent  Present  Absent  Present  Absent  Present  Absent  Present  Absent | | | | | | | | | | | | | | | | | | | |
| **Vocalization:**  **a) Bark**  **b) Whine**  **c) Growl** | | | | | Present  Absent  Present  Absent  Present  Absent | | | | | | | | | | | | | | | | | | | |
| **Pica**  **Coprophagia** | | | | | Present  Absent  Present  Absent | | | | | | | | | | | | | | | | | | | |
| **Handling and Restraint** | | | | | | | | | | | | | | | | | | | | | | | | |
| **Handing** | | | | | | | | | Applied  Not Applied | | | | | | | | | | | | | | | |
| **Restraint** | | | | | | | | | Muzzle  Leash | | | | | | | | | | | | | | | |
| **Water Supply** | | | | | | | | | | | | | | | | | | | | | | | | |
| **Types of drinkers** | | | | | | | | | | Bowl/Bucket  Automatic  Other | | | | | | | | | | | | | | |
| **Is the water sufficient for dogs?**  **Dog should drink 30-50ml per day per kg of bodyweight.**  **Evaluate:** | | | | | | | | | | Yes  No | | | | | | | | | | | | | | |
| **Feeding** | | | | | | | | | | | | | | | | | | | | | | | | |
| **Types of diet** | | | | | | | | Dry Pellets  Wet/Canned  **Meat**  Fresh Meat  Dead Meat  Raw Meat  Cooked meat  Minced Meat  **Chicken**  Fresh Chicken  Dead Chicken  Raw Chicken  Cooked Chicken  Minced Chicken | | | | | | | | | | | | | | | | |
| **Evaluate feeding regime:** | | | | | | | | - Adult Male 00 kg to 00 kg once a day. - Adult Pregnant female: 00 kg to 00 kg once a day. - Adult non-Pregnant female:00 kg to 00 kg once a day. - Adult Nursing female: 00 kg to 00 kg once a day0 - Puppies: 00 gm. to 00 gm. once a day. | | | | | | | | | | | | | | | | |
| **Special Diets for sick**  **Describe:**  **Special diets for geriatrics**  **Describe: Is there a fasting day?** | | | | | | | | Yes  No  (Boiled Vegetables Soup/Boiled minced meat).  Yes  No  (Boiled Vegetables Soup)  Yes (one day /week)  No | | | | | | | | | | | | | | | | |
| **Exercise** | | | | | | | | | | | | | | | | | | | | | | | | |
| **Are Dogs Left in an outdoor fenced Area?** | | | | | | | Yes  No  Frequency:  Daily  Weekly  Time: (00 Minutes or More)  When:  morning  afternoon | | | | | | | | | | | | | | | | | |
| **Are the dogs walked on leash by Farm personnel or by volunteers** | | | | | | | Yes  No | | | | | | | | | | | | | | | | | |
| **Is There a training program for employees/ volunteers?** | | | | | | | Yes  No | | | | | | | | | | | | | | | | | |
| **Grooming** | | | | | | | | | | | | | | | | | | | | | | | | |
| **Is there grooming?** | | | | | | Yes  No | | | | | | | | | | | | | | | | | | |
| **How often dogs grooming?** | | | | | | Daily  Weekly | | | | | | | | | | | | | | | | | | |
| **What are the tools used?** | | | | | |  | | | | | | | | | | | | | | | | | | |
| **What are the methods of grooming?** | | | | | |  | | | | | | | | | | | | | | | | | | |
| **Sanitation** | | | | | | | | | | | | | | | | | | | | | | | | |
| **Is there sanitation program?** | | | | | | Yes  No | | | | | | | | | | | | | | | | | | |
| **Disinfection Practices:** | | | | | | | Daily  Weekly  Monthly  Material used: | | | | | | | | | | | | | | | | | |
| **Pest control:**  **Describe:**  **Is there wheels dipping?**  **Is there Foot dipping?** | | | | | | | Yes  No  Frequency:  Methods:  Material used:  Yes  No (Phenol)  Yes  No (Phenol) | | | | | | | | | | | | | | | | | |
| **Vaccinations** | | | | | | | | | | | | | | | | | | | | | | | | |
| **According to the dog age:** | | | | | | 6-8 weeks (DHPP) | | | | | | | | | | | | | | | | | | |
|  |  |  |  |  |  | 10-12 weeks (DHPP)+Leptospira, Lyme Disease, Bordetella | | | | | | | | | | | | | | | | | | |
|  |  |  |  |  |  | 12-24 weeks (Rabies) | | | | | | | | | | | | | | | | | | |
|  |  |  |  |  |  | 12-16 month (DHPP, Rabies) | | | | | | | | | | | | | | | | | | |
|  |  |  |  |  |  | Every year (DHPP+ Leptospira, Lyme Disease, Bordetella | | | | | | | | | | | | | | | | | | |
|  |  |  |  |  |  | Every 3 Year (Rabies) | | | | | | | | | | | | | | | | | | |
|  |  |  |  |  |  | (DHPP) (Vaccines for Adenovirus Hepatitis, Distemper, Parainfluenza, Parvo Virus. | | | | | | | | | | | | | | | | | | |
| **External and Internal parasiticides** | | | | | | | | | | | | | | | | | | | | | | | | |
| **Is There External parasites?**  **Drugs Used:** | | | | | | | Yes  No  Fipronil  Premethrin …spot on  Premethrin …spot on  Premethrin and Pyriproxyfen  ex.Advantex  Topical Spray | | | | | | | | | | | | | | | | | |
| **Is There Internal parasites?**  **Drugs Used:** | | | | | | | Yes  No  Pyrantel  Levamisole  Febantel  Felabendazole  Dorontal plus  Parziquantel  Pancure | | | | | | | | | | | | | | | | | |
| **Transportation** | | | | | | | | | | | | | | | | | | | | | | | | |
| **Methods of transportation** | | | | | | By car  By train  By airplane  By ship | | | | | | | | | | | | | | | | | | |
| **Dogs Reproduce** | | | | | | | | | | | | | | | | | | | | | | | | |
| **Is there a special place for breeding?** | | | | | | Yes  No | | | | | | | | | | | | | | | | | | |
| **From where the farm brings female dogs for breeding?** | | | | | | Inside ☐ Outside | | | | | | | | | | | | | | | | | | |
| **How many days will a female dog let males mount her?** | | | | | |  | | | | | | | | | | | | | | | | | | |
| **Veterinary Information** | | | | | | | | | | | | | | | | | | | | | | | | |
| **Is there a veterinarian?** | | | | | | Yes  No | | | | | | | | | | | | | | | | | | |
| **Frequency of visits** | | | | | | Once /  week  Month | | | | | | | | | | | | | | | | | | |
| **General** | | | | | | | | | | | | | | | | | | | | | | | | |
| **Does the Farm Sell Dogs to the dealers** | | | | | | Yes  No | | | | | | | | | | | | | | | | | | |
| **What is the attitude of the farm employees/volunteers toward my visit?** | | | | | | Positive ☐ Negative | | | | | | | | | | | | | | | | | | |
|  | | **Isolation** | | | | | | | | | | | | |  | | | | | | | | | |
| **Is There an isolation area for dogs?** | | Yes  No | | | | | | | | | | | | | Good  Fair  Poor | | | | | | | | | |
| **Injured** | | Yes  No | | | | | | | | | | | | | Good  Fair  Poor | | | | | | | | | |
| **Bite Cases** | | Yes  No | | | | | | | | | | | | | Good  Fair  Poor | | | | | | | | | |
| **Sick** | | Yes  No | | | | | | | | | | | | | Good  Fair  Poor | | | | | | | | | |
| **Wildlife** | | Yes  No | | | | | | | | | | | | | Good  Fair  Poor | | | | | | | | | |
| **Other** | | Yes  No | | | | | | | | | | | | | Good  Fair  Poor | | | | | | | | | |
| **Is there quarantine area for new dogs?** | | Yes  No | | | | | | | | | | | | | Good  Fair  Poor | | | | | | | | | |
| **Mortality** | | | | | | | | | | | | | | | | | | | | | | | | |
| **Procedures for disposing of dead Dogs:** | | | | | | Burial  Method:  Material:  Incineration | | | | | | | | | | | | | | | | | | |
